# Cancer-Associated Fibroblasts Move and Interact More with Triple-Negative Breast Cancer Cells and Stimulate Their Proliferation in a Hyaluronan-Dependent Manner

**DOI:** 10.3390/cells14211663

**Published:** 2025-10-23

**Authors:** Sz-Ying Hou, Sarah C. Macfarlane, Ariadna Gómez Torijano, Hyejeong Rosemary Kim, Marieke Rosier, Katalin Dobra, Penelope D. Ottewell, Annica K. B. Gad

**Affiliations:** 1 School of Medicine and Population Health, Clinical Medicine, University of Sheffield, Sheffield S10 2RX, UKsarah.macfarlane@crick.ac.uk (S.C.M.); h.r.kim@sheffield.ac.uk (H.R.K.);; 2 Department of Oncology-Pathology, Karolinska Institutet, 171 64 Solna, Sweden; ariadgom@ucm.es (A.G.T.); katalin.dobra@ki.se (K.D.); 3 School of Science and Technology, Örebro University, 701 82 Örebro, Sweden

**Keywords:** cancer-associated fibroblasts, triple-negative breast cancer, 3D spheroid co-culture models, hyaluronan

## Abstract

**Highlights:**

**What are the main findings?**

- Cancer-associated fibroblasts from Triple-negative breast cancer secrete high levels of hyaluronan, compared with normal breast fibroblasts, altering the tumour microenvironment.
- Hyaluronan production by Triple-negative breast cancer-associated fibroblasts enhances mixing of Triple-negative breast cancer cells with fibroblasts and promotes progression of this aggressive cancer type.

**What is the implication of the main finding?**

- Inhibition of the production of hyaluronan by Triple-negative breast cancer-associated fibroblasts is a potential future therapeutic target against Triple-negative breast cancer progression.

**Abstract:**

While normal fibroblasts suppress tumor growth, during cancer initiation and progression, this capacity can be lost and even switched to tumor-promoting, for reasons that are not understood. In this study, we aimed to determine differences between patient-derived cancer-associated fibroblasts and fibroblasts from healthy breast tissue to identify if and how these changes stimulate Triple-negative breast cancer (TNBC). Two-dimensional and three-dimensional mono and co-cultures of TNBC cells with fibroblasts from healthy breast or TNBC were analyzed for cell contractility, migration, distribution, proliferation, and hyaluronan production by traction force microscopy, live cell imaging, flow cytometry, Western blot, and ELISA. In 3D spheroid co-culture, CAFs migrated into the tumor mass, mixing with tumor cells, whereas normal fibroblasts remained separate. In 2D, CAFs showed increased cell migration and contractile force, and, in both 2D and 3D co-culture, CAFs increased the proliferation of TNBC cells. CAFs showed increased production of hyaluronan, as compared to normal fibroblasts, and loss of hyaluronan synthase 2 reduced CAF-induced stimulation of TNBC proliferation. These findings suggest that increased production of hyaluronan by TNBC CAFs enhances their capacity to mix with and induce the proliferation of cancer cells, and that the production of hyaluronan by CAFs can be a future therapeutic target against TNBC.

## 1. Introduction

Breast cancer is the most diagnosed cancer and the leading cause of cancer-related death in women worldwide [1]. Triple-negative breast cancer (TNBC) is a subtype of breast cancer defined by the absence of the estrogen and progesterone receptors, and of the human epidermal growth factor receptor 2 (HER2) [2]. TNBC accounts for approximately 8–25% of breast cancer cases and is linked to a higher incidence of early recurrence, distant metastases, and a poorer prognosis than other subtypes of breast cancer [3]. The 5-year overall survival for metastatic TNBC is around 11%, with a median overall survival of around 11 to 13 months [4,5]. There is therefore an urgent need to understand the molecular mechanisms that cause the disease and identify novel approaches to block the growth and spread of TNBC.

In comparison to other breast cancer subtypes, TNBC frequently shows high mammographic density and a well-defined, smooth, pushing border between the tumour and surrounding stroma. TNBC lacks the typical malignant features of breast cancer, such as invasive and spiculated margins. The circumscribed and pushing tumour margin is found in aggressive tumours, in which the high proliferation rate of the cancer cells pushes them towards the stroma [6]. Accordingly, this phenotype is linked to increased nuclear pleomorphism and higher tumour grade, in comparison to tumours with spiculated margins [6,7,8]. In TNBC, the ratio between the tumour cells of epithelial origin and the tumour stroma is a prognostic factor [9]; a TNBC with more than 50% stroma has a shorter relapse-free period and lower overall survival than a TNBC with less stroma [10,11]. The TNBC stroma has also been found to be mechanically stiffer than that of other types of breast cancer [12]. Taken together, this can suggest that the TNBC stroma promotes the growth and spread of the tumour. On the other hand, it has been suggested that, as TNBC is a very fast-growing tumour type, it might not have the time required to promote stromal interactions, and that the high stroma content would rather be a consequence and not a cause of the disease [6]. Fibroblasts are amongst the most abundant cells in the stroma, in which they produce and remodel the extracellular matrix (ECM). Thereby, the cancer-associated fibroblasts (CAFs) control the composition of the stroma, along with the mechanical stiffness and the physical forces therein. Cells can sense and respond to the physical and mechanical properties around them [13], and this capacity is increased, and even mimicked by the presence of hyaluronan in the environment [14]. Hyaluronan is a non-sulfated glycosaminoglycan that is synthesised by hyaluronan synthases (HAS) at the cell border, and is widely present in the ECM. Hyaluronan and the downstream intracellular signalling is initiated by the binding of hyaluronan to cell surface receptors which is important for tumor development and progression [15,16,17,18,19,20]. It has been found to stimulate breast cancer progression [18,19,20], and is further increased in the stroma of TNBC, as compared to that of other breast cancer types [21]. The binding to hyaluronan further causes similar effects in the cell as a stiff extracellular environment [14]. We therefore hypothesized that the production of hyaluronan by CAFs in TNBC promotes the growth and spread of the tumour.

In this manuscript, we present new data suggesting that CAFs from TNBC patients increase the production of hyaluronan and exert increased cellular contractile force, resulting in increased movement, mixing, and interaction with TNBC cells, stimulating their proliferation, in a hyaluronan-dependent manner. In summary, these findings present novel avenues for future approaches to treat TNBC, based on the capacity of CAFs to produce hyaluronan.

## 2. Materials and Methods

### 2.1. Cell Lines and Cell Culture

The CAF subtypes 2804T and 2262T were derived from the surgical resection of TNBC from 46- and 56-year-old TNBC patients, diagnosed with grade II and III invasive ductal carcinoma, respectively. Representative histological sections of 2804T and 2262T can be seen in Supplementary Figure S1. The NFs 3045N and Ls14 were derived from 55- or 60-year-old healthy women, respectively, following breast reduction surgery. All fibroblasts were obtained from Breast Cancer Now, UK, which identified and validated the cells to be fibroblasts, along with their standard procedures. The fibroblasts were cultured in DMEM/F12 (Lonza, Basel, Switzerland) containing L-glutamine (Lonza, Switzerland) with 10% fetal bovine serum (Sigma Aldrich, Dorset, UK), 100 U/mL penicillin, and 100 µg/mL streptomycin (Lonza, Switzerland). The human TNBC cell line HCC1143 (ATCC, Manassas, VA, USA) was cultured in RPMI (Lonza, Switzerland) containing L-glutamine with 10% fetal bovine serum, 100 U/mL penicillin, and 100 µg/mL streptomycin. All cells were maintained at 37 °C, 5% CO_2_. Fibroblasts were used for experiments at passage 7 or below.

### 2.2. Western Blot

Primary antibodies included anti-vimentin, B-actin (a1978, Sigma-Aldrich, USA), HAS2 (sc-514737, Santa Cruz, Dallas, TX, USA), α-SMA (ab124964, Abcam, Cambridge, UK), FAP-α (ab28244, Abcam, UK), and GAPDH (sc-32233, Santa Cruz, USA). Secondary antibodies included anti-mouse Alexa 488 (A32766, Thermo Fisher, Waltham, MA, USA), anti-guinea pig Alexa 555 (ab150188, Abcam, UK), and anti-mouse Alexa 647 (A21236, Thermo Fisher, USA). Media was aspirated and cells were washed twice with ice-cold PBS. Cells were lysed using 15 µL of RIPA buffer per 1 cm^2^ on ice for 10 min. A cell scraper was used to remove all cells, which were transferred to an Eppendorf tube on ice and incubated for 10 min. Samples were vortexed twice for 15 s and centrifuged at 10,000 rpm for 5 min at 4 °C. Pellets were discarded, and supernatants were transferred to new tubes. Samples were stored at −80 °C until needed. Protein concentration was quantified via bicinchoninic protein assay according to the manufacturer’s protocol. Samples were prepared by diluting 3:1 in 4× Laemmli buffer containing 50 mM dithiothreitol. Samples were boiled for 5 min on a heat block at 90 °C. Immediately after boiling, samples were centrifuged at 3000 rpm for 1 min. Samples and protein ladder were loaded onto 4–15% precast gels and run in 1× TGS running buffer at 100 volts for approximately 1 h. Samples were transferred from the gel onto a nitrocellulose membrane using the Trans-Blot Turbo Transfer System (Bio-Rad, Hercules, CA, USA) on the mixed molecular weight setting, which ran at 25 volts for 7 min. The membrane was stained with Ponceau S solution to confirm a successful transfer and cut if necessary. Membranes were blocked in TBST containing 5% milk (as standard) or 5% bovine serum albumin (BSA) (for HAS2 or phosphorylated proteins) for 1 h on a shaker at room temperature. Primary antibodies were diluted in TBST containing 5% milk or 5% BSA and incubated with the membrane overnight at 4 °C on a roller. The following day, membranes were washed twice quickly, then washed 3 × 10 min in TBST on a shaker. Secondary antibodies were diluted in TBST containing 5% milk or 5% BSA and incubated for 1 h at room temperature on a shaker. The membrane was washed twice quickly, then washed 3 × 10 min in TBST on a shaker protected from light. After being covered with HRP substrate, the membranes were visualised using the ChemiDoc Imaging system (Bio-Rad, USA). The signal intensity of the bands was quantified in FIJI Version 2.3 [22]. Each sample was normalised to the corresponding β-actin or GAPDH loading control band. If necessary, membranes were stripped and re-probed. After visualising the first protein of interest, membranes were washed for 3 × 10 min in TBST at room temperature and then incubated with 10 mL stripping buffer at 37 °C for 10 min. To confirm that there was no remaining signal, the membranes were washed for 2 × 10 min in TBST and imaged with HRP substrate. If any signal remained, the stripping process was repeated. If no signal remained, membranes were washed for 2 × 10 min in TBST, and blocking, antibody staining, and visualisation were repeated with the next antibodies.

### 2.3. Traction Force Microscopy

Collagen I-coated hydrogels of 12 kPa containing 0.2 µm green fluorescent beads (Cell Guidance Systems, Cambridge, UK) were incubated for 30 min at 37 °C with media. Thereafter, 10,000 cells were seeded per gel and incubated for 5 h. Images of the beads under the attached cells were taken, then cells were detached by 5 min incubation in 0.5% (*v*/*v*) Triton X-100 and 20 mM NH_4_OH in PBS, followed by imaging the beads in the same area. Bead displacement was measured using particle image velocimetry, followed by Fourier transform traction cytometry using a custom macro kindly shared by Dr Stacey Lee [23], from a previously described method [24]. The contractile force of single cells was analysed in FIJI, as described previously [25].

### 2.4. Cell Migration Assay

Fibroblasts were seeded in CELLview cell culture slides at 1000 cells per well and incubated for 24 h. Thirty minutes prior to imaging, the media was then replaced with fresh media containing 0.05 μg/mL Hoechst (H3570; Invitrogen, Carlsbad, CA, USA). Cells were maintained at 37 °C and 5% CO_2_ and imaged every 45 min for 15 h.

### 2.5. Ex Vivo 2D Co-Culture Model of TNBC Cells and CAFs

HCC1143 cells were seeded in CELLview cell culture slides (Grenier Bio-One, Austria, Germany) at a density of 5 × 10^4^ cells per well, and incubated for 3 days in media containing Cell Tracking Red Dye Kit (Abcam, UK) at a dilution of 1:20. On day 3, a confluent cell monolayer was formed. We then created cancer cell clusters of approximately 2500 HCC1143 cells per cluster by removing cells with a P1000 pipette tip, followed by 3 washes in PBS. Thereafter, 1 × 10^4^ fibroblasts were seeded per well and incubated for 24 h. Cell viability was assessed by incubating co-cultures with medium containing 0.05 µg/mL Hoechst and 1 µg/mL propidium iodide for 30 min and calculating the percentage of propidium iodide-positive nuclei.

### 2.6. Ex Vivo 3D Co-Culture Model of TNBC Cells and CAFs

To fluorescently label the cancer cells and fibroblasts, HCC1143 cells were treated with media containing Cell Tracking Red Dye Kit at a dilution of 1:20 for 24 h, and fibroblasts were infected with 400 infectious units per cell of GFP-adenovirus for 24 h, as described previously [26]. We then created co-culture spheroids using the liquid overlay technique by seeding cancer cells and fibroblasts in a ratio of HCC1143-fibroblasts of 1:5, i.e., 4000 HCC1143 and 16,000 fibroblasts, or 4000 HCC1143 only in 96-well low attachment U-bottom plates (Thermo Fisher, USA), followed by a 72 h incubation. To allow imaging, we transferred the spheroids from the U-bottom plates to CELLview cell culture slides. Due to the risk of this procedure introducing inter-personal variations to the spheroid shape as a consequence of the force applied when pipetting, the spheroids with fibroblasts were compared to the control spheroid created by the same person.

### 2.7. Microscopy and Image Analysis

Paraffin-embedded TNBC tissue sections were stained with hematoxylin and eosin and imaged on a NanoZoomer slide scanner (Hamamatsu Photonics, Hamamatsu City, Japan). The 2D and 3D models were imaged with a Cell Discoverer 7 (Zeiss, Oberkochen, Germany). Live imaging was performed at 37 °C, 5% CO_2_. Images taken every 40 min with a 20× objective with the Nikon CrEST X-Light V3 (Nikon Corporation, Tokyo, Japan), followed by analysis in FIJI using the TrackMate plugin, as described earlier [23]. For analysis of the distribution of cells within spheroids, Z-stacks of green (fibroblasts) or red (cancer cells) fluorescent channels were captured with Cell Discoverer 7 using the Zeiss Zen software (version 3.2, Zeiss, Germany), followed by computational analysis in FIJI. Briefly, we created a maximum intensity projection in FIJI, and then applied a binary mask to identify centroids, using a custom macro (Supplementary Figure S2 and Text S1), followed by extraction of positions and nearest neighbor distance analysis of spheroids, using a home-written Python 3.9.21 script based on the SciPy library (spatial. KDTree) algorithm (Supplementary Text S2).

### 2.8. Flow Cytometry

Spheroids from technical replicates were pooled and incubated with Accutase (Thermo Fisher, USA) under agitation at 37 °C for 20 min, followed by a wash in 1% BSA/PBS by centrifugation. The cells were then resuspended in 1% BSA/PBS, passed through a cell strainer cap into flow cytometry tubes (Corning Inc., Corning, NY, USA), and analysed with a BD LSRII flow cytometer using the BD FACSDiva Software (version 6.1.3, BD Biosciences, Bedford, MA, USA). To analyse viability, samples were dyed by adding 1 µM TO-PRO3 (Thermo Fisher, USA) just before running. Samples were run on an LSRII flow cytometer (BD Biosciences, USA) and analysed or sorted by size and signal intensity. Gating and analysis were performed using FlowJo software (version 10.8, BD Biosciences, USA).

### 2.9. Immunofluorescence Staining

2D samples were fixed and stained as previously described [27]. Primary antibodies included anti-vimentin (V6389, Sigma Aldrich, USA) and anti-pan-cytokeratin (BP5069, Origene, Rockville, MD, USA). Secondary antibodies included anti-mouse Alexa 488 (A-11001, Thermo Fisher, USA), anti-guinea pig Alexa 594 (ab150188, Abcam, UK), and anti-mouse Alexa 647 (A-21235, Thermo Fisher, USA). Other fluorescent stains included Phalloidin-Alexa 488 and DAPI.

### 2.10. Blebbistatin Treatment

Samples were treated with 50 µM of Blebbistatin (Sigma-Aldrich, USA). A 10 mg/mL stock solution was diluted 1:1000 in media to obtain a working concentration of 10 µg/mL. DMSO was used as the vehicle control at a 1:1000 dilution. Cells were incubated with Blebbistatin or DMSO for 72 h prior to analysis.

### 2.11. Transient Knockdown of HAS2

For HAS2 knockdown in CAF cells, we used HAS2-targeting ON-TARGET siRNA-SMART pool (J-012053-21-0020, Dharmacon, Lafayette, CO, USA) or Non-targeting Control siRNA pool (D-001810-10-20, Dharmacon) as a control. These pools consisted of a mixture of 4 separate siRNAs each. These mixtures were transfected using siLentFect^TM^ INTERFERin transfection reagents for RNAi (1703361, Bio-Raed, Hercules, CA, USA) according to the manufacturer’s protocol.

### 2.12. Proliferation Assay

Cells sorted with flow cytometry were collected on an Epredia Cytoslide microscopy slide (Thermo Fisher Scientific, Waltham, MA, USA), using a Shandon Cytospin 3 Cytocentrifuge (Thermo Fisher Scientific), followed by immunofluorescence staining of Ki67 (ab16667, Abcam, Cambridge, UK), as described above. The ratio of Ki67-positive cancer cells to all cancer cells was then determined.

### 2.13. Two-Dimensional Co-Culture Viability

After 72 h of co-culture, the media was changed to fresh phenol red-free media containing 1 µg/mL propidium iodide and 0.05 µg/mL Hoechst, followed by a 30 min incubation prior to imaging using a Cell Discoverer 7 microscope at 37 °C, 5% CO_2_.

### 2.14. Generation of Fibroblast-Derived ECM

Fibroblasts were seeded onto coverslips coated with gelatin, as follows: 13 mm diameter glass coverslips were placed into a 24-well plate. A total of 200 µL of 0.2% gelatin in H_2_O (Sigma-Aldrich, USA) was added to each well and incubated for 1 h at 37 °C. Gelatin was aspirated and wells washed once with PBS and incubated with 200 µL of 1% glutaraldehyde for 30 min at room temperature. Glutaraldehyde was aspirated, and wells were washed for 3 × 5 min with PBS. Then, 200 µL of 1 M glycine was added to each well and incubated for 20 min at room temperature, followed by another 3 × 5 min wash in PBS. Cells were thereafter seeded at a density of 10,000 cells per cm^2^ in complete media and incubated for 24 h. At this stage, siRNA transfection was performed. After 24 h, the media was changed to fresh media containing 50 µg/mL ascorbic acid (Sigma-Aldrich, USA) and incubated for a further 72 h. The matrices were decellularized by washing once in warm PBS, followed by 200 µL of warm (37 °C) cell extraction buffer (20 nM NH_4_OH, 0.5% Triton X-100 in PBS) for 5 min at 37 °C. Cell lysis was confirmed by checking under a brightfield microscope. The matrix was washed 3 times very gently with PBS to remove all cell debris. The resulting decellularized matrices were used immediately for cell seeding, followed by a 48h incubation and fixation for immunofluorescence staining.

### 2.15. ECM Alignment and Coherency

ECM alignment and coherency were analysed using the OrientationJ plugin in FIJI [28].

### 2.16. Statistical Analysis

All data is generated from at least 3 biological, experimental repeats. Statistical analysis was performed in GraphPad Prism (version 9.1.0). Data were tested for normality using the D’Agostino and Pearson test, followed by an unpaired *t*-test for parametric data, or the Mann–Whitney test for non-parametric data. The fluorescent signal of spheroid maximum intensity projections was analysed using a 2-way ANOVA.

## 3. Results

### 3.1. Triple-Negative Breast Cancer Fibroblasts Are More Elongated than Normal Breast Fibroblasts and Show Increased Cellular Contractile Force, Cell Migration Speed, and Persistence

**Figure 1:**
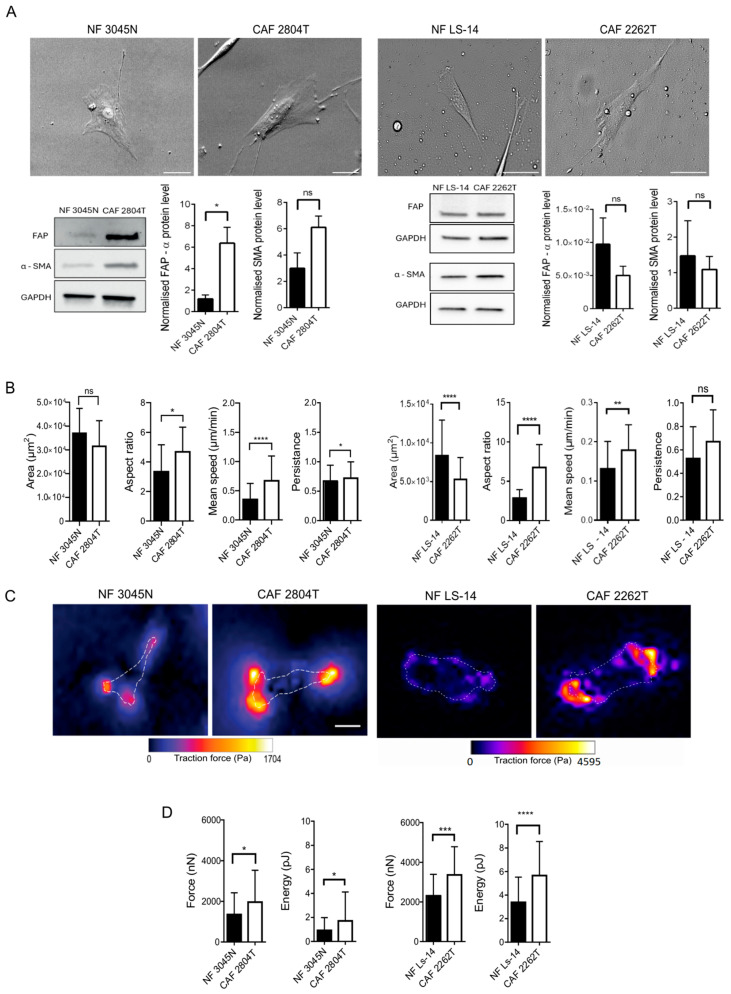
Cancer-associated fibroblasts from TNBC are more elongated, more migratory, and exert increased cellular contractile force as compared to fibroblasts from healthy breast tissue. (**A**) Representative images of NFs and CAF, as indicated. Scale bar: 50 µM. Representative Western blot membranes showing myofibroblast markers (FAP, SMA) with GAPDH as a loading control. * = *p* > 0.05, ns = not significant. (**B**) Area, aspect ratio, mean speed, and mean persistence of cells, with graphs showing mean ± SEM of at least three independent biological repeats, with *n* ≥ 139 cells per condition, analysed with a Mann–Whitney test. * = *p* > 0.05, ** = *p* < 0.01, **** = *p* > 0.0001, ns = not significant. (**C**) Representative heat maps of the traction force exerted in nNand pJ by the indicated NFs and CAFs. The dashed white line indicates the outline of a single cell. Scale bar: 40 µM. (**D**) Quantification of the energy and force, showing the mean ± SEM of at least four independent biological repeats of the following number of cells (in brackets): CAF 2804T (≥35), CAF 2262T (≥40), NFs 3045N (≥40), and Ls14 (≥50). Data were analysed using either a Mann–Whitney test or an unpaired *t*-test, depending on data normality. * = *p* < 0.05, *** = *p* < 0.001, **** = *p* < 0.0001.

To confirm that the cells we had received were fibroblasts and CAFs, we compared the levels of fibroblast activation protein alpha (FAP-α), as well as of alpha-smooth muscle actin (αSMA), the latter a common marker to distinguish CAFs from NF. While the cancer-associated fibroblasts from one TNBC patient showed increased levels of FAP-α and a trend towards increased αSMA, as compared to fibroblasts from healthy breast tissue, cancer-associated fibroblasts from another TNBC patient did not show the same results (Figure 1A). The mesenchymal marker vimentin was highly expressed in fibroblasts, while keratin, an epithelial marker, was present only in the epithelial cancer cells (Supplementary Figure S3B). Taken together, this confirms that the patient-derived cells obtained were fibroblasts. To further identify properties of the CAFs that could promote cancer, we analysed the morphology, shape, and capacity of the fibroblasts to move on a 2D surface, using live cell imaging. The TNBC CAFs derived from both patients showed a more elongated morphology, along with a trend towards increased speed and persistence of cell migration, as compared to NF (Figure 1B), (Supplementary Figure S4). αSMA expression indicates an activated myofibroblast CAF phenotype, which results in an increased capacity of the cells to migrate and exert cellular contractile force on their surrounding environment. While we did not observe increased levels of αSMA in CAFs, protein markers for CAFs are not definitive, and we reasoned that an increased cellular contractile force of the CAFs, as compared to NFs, would be the best indicator that the fibroblasts from the TNBC were CAFs. We thereafter analysed the contractile force of the cells with traction force microscopy. We observed that both CAF variants exerted an increased cellular contractile force on 12 kPa hydrogels, as compared to NF (Figure 1C). Taken together, these findings highlight that the fibroblasts derived from TNBC tissue are CAFs, which show increased elongated cell shape, motility, and cellular contractile force.

### 3.2. TNBC CAF Co-Culture Reduces the Size of TNBC Cancer Cell Clusters in 2D

**Figure 2:**
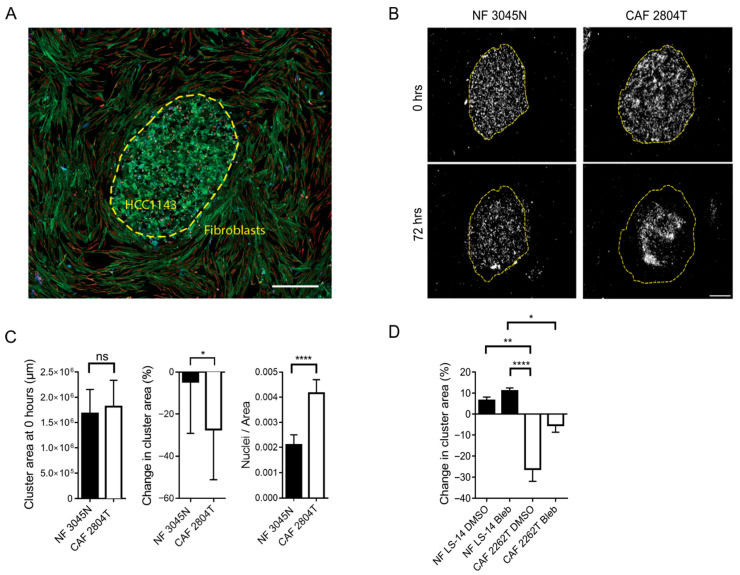
**Two-dimensional co-culture of CAFs (2804T) compresses cancer cell clusters.** (**A**) Representative image of 2D co-culture of a cluster of HCC1143 with surrounding fibroblasts, as indicated (yellow dashed line), showing F-actin (green), pan-cytokeratin (blue), and vimentin (red). Scale bar: 500 µm. (**B**) Representative cancer cell clusters in 2D co-culture with normal fibroblasts (NF) (top) or CAFs (lower panel) at 0 (left) and 72 h (right panel), showing pan-cytokeratin positive cancer cells (white), with the cluster boundary at 0 h indicated by a yellow dashed line. Scale bar: 500 µm, as quantified in (**C**), showing the change in cluster area (%) during the experiment, the initial area and nuclei density of the clusters, and the cluster area at 0 h. The nuclei density in the clusters and cluster area at 0 h show the mean +/− SEM of three independent biological repeats, with CAF *n* ≥ 12 2D samples analysed per condition. Change in cluster area and cluster area at 0 h were analysed using a Mann–Whitney test, and nuclei/area were analysed using an unpaired *t*-test. (**D**) changes in cancer cluster area in co-culture with NF or CAFs, upon treatment with Blebbistatin (Bleb) or DMSO control. * = *p* < 0.05, ** = *p* < 0.01, **** = *p* < 0.0001, ns = not significant.

We could observe a circumscribed morphology with a clear boundary between the tumour and stroma in sections of the tumours from which the CAFs had been isolated (Supplementary Figure S1). Peripheral to the tumour core, TNBC cells grew in the stroma, in circumscribed islets or clusters with an average diameter of 150 μm. To establish a model system in which we could study the effect of fibroblasts at the tumour-stroma interface, we created an ex vivo model in which the cancer cells grew in circumscribed 2D cell clusters, with or without a surrounding monolayer of either CAFs or NFs (Figure 2A). In the presence of fibroblasts, we observed that over 72 h, the area of HCC1143 clusters decreased (Figure 2B,C), while cell density within the clusters increased, with no effect on cell proliferation or viability (Supplementary Figures S5 and S6). This occurred to a greater extent in co-culture with CAF than in co-culture with NFs (Figure 2C). To further determine if acto-myosin-based contractile force was required for the observed effect on cancer cell clusters, we treated the co-cultures with the specific myosin inhibitor Blebbistatin. To this end, we observed that Blebbistatin reduced the difference in cluster size observed between co-culture NFs or CAFs (Figure 2D). Taken together, this data suggests that CAFs may regulate cancer cells by exerting force that compresses TNBC cell clusters.

### 3.3. TNBC CAFs Mix More with Cancer Cells and Show Reduced Capacity to Suppress the Growth of Cancer Cells in 3D, Compared with NFs

**Figure 3:**
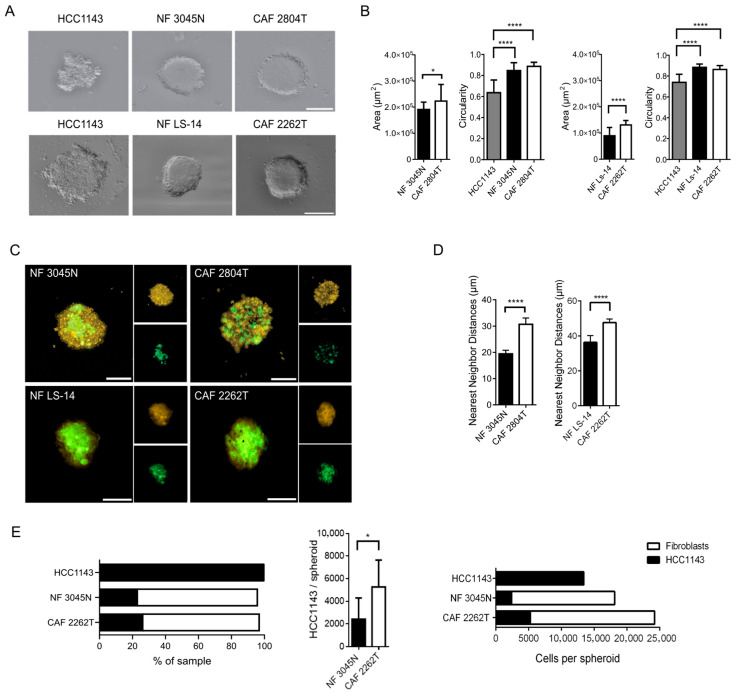
**Triple-negative breast cancer–associated fibroblasts show increased capacity to mix with cancer cells in 3D.** (**A**) Representative images of HCC1134 TNBC cancer cells 3D spheroids seeded with HCC1143 cancer cells only, or in co-culture with the NFs 3045N or LS-14, or the CAFs 2804T or 2262T, as indicated. Scale bar: 300 µm. (**B**) Quantification of area and circularity in each 3D co-culture condition, showing the mean ± SEM from 3 independent biological repeat experiments with *n* ≥ 23 spheroids per condition, with circularity analysed with a Kruskal–Wallis test and Dunn’s multiple comparisons test and area analysed with a Mann–Whitney test. * = *p* < 0.05, **** = *p* < 0.0001. (**C**) Immunofluorescence images of the co-culture spheroids composed of HCC1143 in co-culture with the NFs 3045N or LS-14, or the CAFs 2804T or 2262, as indicated, showing HCC1143 cancer cells (yellow) and fibroblasts (green), with corresponding single channels (right). Scale bar: 200 µm, with (**D**) the spatial distribution in 3D analysed with nearest neighbour analysis for the spheroids (number total spheroids indicated in brackets), HCC1134 with CAF 2804T (7), CAF 2262T (15), NF Ls-14 (9), or NF 3045N (6), using the Mann–Whitney U test (****, *p* < 0.0001). (**E**) Fraction and number of HCC1143 (black) and fibroblasts (white) per spheroid, as indicated. Data show mean, error bars represent SEM. Data were obtained from three independent biological repeats with *n* ≥ 23 spheroids per condition. HCC1143/spheroid was analysed using a paired *t*-test. * = *p* < 0.05.

To better mimic the spatial properties of the cancer in the tissue, we established an ex vivo 3D spheroid model in which HCC1143 cells were cultured with or without NF or CAF. We observed that if the HCC1143 cells were cultured alone, the spheroids formed were irregularly shaped, fragile, and often disintegrated during handling, whereas the co-culture spheroids were compact, stable, and had uniform boundaries. CAF co-culture spheroids grew larger with higher circularity than NF co-culture spheroids (Figure 3A,B). To gain insights into how fibroblasts can regulate cancer growth, we then determined the spatial distribution of each cell type in the spheroids. While the HCC1143 showed a uniform distribution throughout the spheroids, regardless of whether they were co-cultured with NFs or CAFs, the fibroblasts showed distinct distribution patterns. CAFs were diffused throughout the spheroids, quantified as increased distance from their nearest-neighbour, whereas NF showed a more aggregated and clustered distribution (Figure 3C,D). No effect on fibroblast or cancer cell viability was observed (Supplementary Figure S7). To determine if 3D co-culture with CAFs promotes the growth of cancer cells, we then analysed the fraction of the HCC1143 cells in the spheroids with or without the CAF or NFs using flow cytometry. Co-culture with fibroblasts reduced the number of HCC1143 cells compared to monoculture. In co-culture spheroids, co-culture of HCC1143 cells with CAF resulted in more than a two-fold increase in HCC1143 cells as compared to co-culture spheroids containing NF (Figure 3E). In addition, we observed that CAFs induced the proliferation of cancer cells (Supplementary Figure S8). Taken together, these data suggest that fibroblasts suppress the growth of HCC1143 cells in 3D co-culture, and that this suppressive effect is reduced in the presence of CAFs.

### 3.4. Increased Production and Deposition of Hyaluronan by TNBC CAFs Was Required for Cancer Cell Proliferation in 3D Spheroids

**Figure 4:**
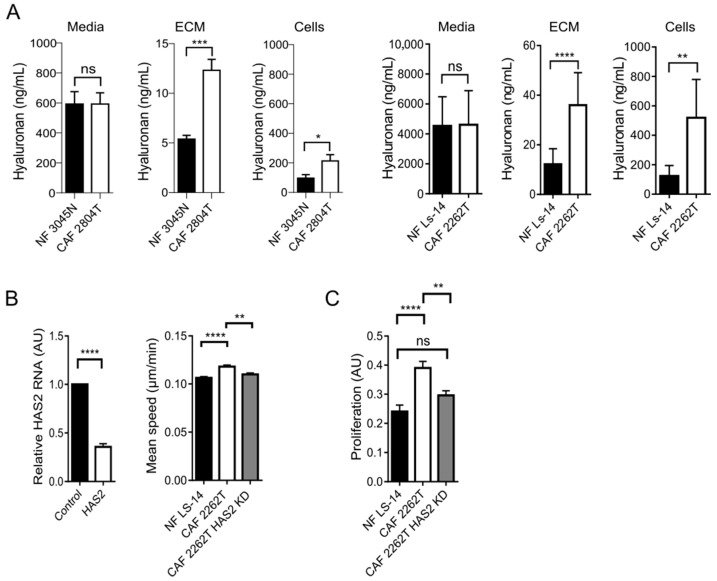
**The increased production of hyaluronan is required for the capacity of Cancer-associated fibroblasts to stimulate the proliferation of cancer cells.** (**A**) Hyaluronan content of conditioned media, cells, and ECM in NF and CAF cells is indicated, with quantification showing the mean +/− SEM of 3 independent biological repeat experiments with 2 technical replicates for each sample. (**B**) Ki67-positive HCC1143 cells of total HCC1143 cells (>3000) (left), after treatment with control or HAS2 siRNA (right panel). (**C**) mean speed of cells, with graphs showing mean ± SEM of at least three independent biological repeats, with *n* ≥ 450 cells per condition. The data was analysed using either a Mann–Whitney test or an unpaired *t*-test, depending on data normality. * = *p* < 0.05, ** = *p* < 0.01, *** = *p* < 0.001, **** = *p* > 0.0001, ns = not significant.

We then aimed to understand why CAFs showed a more heterogeneous distribution in the 3D spheroids. Hyaluronan is one of the most abundant components of the ECM, and it has previously been found to regulate many aspects of cancer [15,16,17]. Therefore, we hypothesized that an increased production of hyaluronan in CAFs could promote the spread of cells. We quantified and compared the levels of soluble, intracellular, and extracellularly deposited hyaluronan produced by CAF and NF. We observed that while the level of soluble hyaluronan in the media did not differ between NFs or CAFs, the levels of intracellular hyaluronan and insoluble hyaluronan deposited into the extracellular matrix were significantly increased in CAFs (Figure 4A). The increased deposition of hyaluronan into the ECM was also visible in the 2D co-culture model (Supplementary Figure S9). Hyaluronan synthase 2 (HAS2) is responsible for most of the production of hyaluronan [29], and it is likely the most prominent HAS in fibroblasts [17,30]. Furthermore, the mRNA levels of HAS2 are significantly higher in TNBC cell lines than in cell lines from less aggressive ER+ breast cancer [31]. To understand the functional role of hyaluronan in TNBC, we therefore knocked down HAS2 and analysed the effect on the CAFs and the TNBC cancer cells. We observed that HAS2 knockdown reduced the migration speed of CAFs to that of normal fibroblasts (Figure 2B and Figure 4B). The level of hyaluronan that the CAFs deposited into the extracellular matrix was further reduced upon loss of HAS2 (Supplementary Figure S10), and this matrix became less coherent (Supplementary Figure S11). We observed no effect on the proliferation of cancer cells on the hyaluronan-deficient matrix (Supplementary Figure S12). However, matrix induced a less spread, and more elongated shape of the cancer cells (Supplementary Figure S13). In 3D co-culture, CAFs stimulated the proliferation of cancer cells, and this effect was lost upon HAS2 knockdown (Figure 4B). Taken together, these findings suggest that hyaluronan produced by TNBC CAFs regulates TNBC cancer cells and that it might have a key role in the maintenance of TNBC.

## 4. Discussion

In this study, we aim to understand the role of the physical and contractile properties of CAFs and the ECM they produce in TNBC. Given the abundance and unique mechanical and architectural characteristics of the TNBC stroma, such as increased ECM stiffness and a distinct tumour-stroma boundary, the physical properties of the stroma have been suggested to be as important as paracrine signaling for the progression of the disease [6,10,11,32]. To allow detailed studies of the TNBC-stroma interface and the role of fibroblasts in TNBC, we developed ex vivo 2D and 3D models composed of fibroblasts derived from patients and healthy individuals in co-culture with TNBC cancer cells. Because TNBC shows a clear, circumscribed, and pushing tumour margin, and a high interstitial pressure, as detected by a significant expansion towards a cut through the tumour (personal communication with a surgeon), we hypothesised that in 2D, the CAFs would compress the cancer cells. In line with this hypothesis, we observed that the CAFs reduced the area of the cancer islets with time, a finding consistent with previous observations of compression of TNBC cells by CAFs in 2D [33]. In the 2D co-culture model, cancer cells and CAFs grew in monolayers on glass, which allows for high-resolution imaging of live and fixed samples. However, as the underlying glass is far stiffer than the tumour microenvironment and cells in 2D culture lack physiological 3D cell-cell contact, we also developed an ex vivo 3D model in which the cells were co-cultured in 3D spheroids. To ensure that only the ECM secreted by the cells would influence their behaviour, we did not include extraneous, non-human ECM components commonly used to produce spheroids, such as Matrigel and rat or bovine collagen I. In the spheroids, the distribution of CAFs was more homogenous than the NFs, which instead predominantly localised in one or a few distinct areas. We speculate that this could be due to the increased capacity of the CAFs to move and/or to bind to the TNBC cancer cells, for example, through adhesions between CAF N-cadherin and cancer cell E-cadherin [34], or by altered ECM deposition and remodeling [35,36,37]. The CAFs from TNBC exerted a higher level of cellular contractile force, as compared to NFs. Our observations are in line with the previous findings that fibroblasts from TNBC cancer tissue have an increased capacity to contract the environment with increased disease stage [38]. Because the contractile force that cells exert on their surrounding environment has been found to be positively correlated to their capacity to move and invade [39], it is possible that these contractile and motile phenotypes indicate an increased capacity for CAFs to invade into their surrounding environment. Previous findings have shown that human CAFs can use actomyosin-based contractile force to compress colorectal cancer cells [33]. However, the consequence of the compression of CAFs on epithelial cancer cells remains to be fully understood.

We observed that CAFs produced increased levels of hyaluronan and stimulated the proliferation of nearby epithelial TNBC cancer cells, enhanced by the main hyaluronan synthase, HAS2. This suggests that hyaluronan promotes the growth and/or spread of cancer, and is in line with previous findings that high levels of hyaluronan in the cancer stroma are linked to poor survival in breast cancer [20,40,41], as well as in numerous other cancer types [42,43,44]. The finding that a small rodent, the naked mole rat (*Heterocephalus glaber*), is highly resistant to cancer, due to a loss of capacity to degrade high to low molecular weight hyaluronan [45,46] suggests that it is the low molecular weight variants of hyaluronan that stimulate cancer. However, the observation that TNBC show, along with high levels of HAS2, increased levels of a hyaluronidase, indicates that the regulation in mole rat and TNBC might differ. In line with this concept, we have previously observed that an increased level of hyaluronan, along with an increased spatial clustering of the molecule at the nanoscale level, is linked to increased fibroblast motility and fibrosarcoma [47]. To our knowledge, our findings are the first to show a functional role of HAS2 in TNBC. Further studies should investigate whether hyaluronan promotes the motility of CAFs by knocking down the downstream stream hyaluronan scaffold protein IQGAP1, which regulates the motility of fibroblasts [48], and the hyaluronan receptors CD44 and RHAMM [49].

In summary, we have used fibroblasts from TNBC patients and healthy individuals to develop 2D and 3D ex vivo model systems that recapitulate features of the interactions between human TNBC cells and the surrounding fibroblasts. Using these systems, we found that the patient-derived TNBC CAFs show an increased capacity to compress, move, mix, interact with, and stimulate the proliferation of TNBC cancer cells, as compared to normal fibroblasts from healthy breast tissue, and that hyaluronan can have a key role in CAF-dependent stimulation of TNBC.

## 5. Conclusions

The findings show, for the first time, that the production of hyaluronan by TNBC CAFs enhances their capacity to move, mix with, and induce the proliferation of TNBC cancer cells, and suggest hyaluronan production by TNBC CAFs as a therapeutic target against TNBC.

## Data Availability

The original contributions presented in this study are included in the article/Supplementary Materials. Further inquiries can be directed to the corresponding author.

## Supplementary Materials

The following supporting information can be downloaded at: https://www.mdpi.com/article/10.3390/cells14211663/s1, Figure S1: Representative histological section of triple negative breast cancer; Figure S2: Image acquisition and cell position extraction using FIJI; Figure S3: Western blot characterisation of fibroblast and myofibroblast markers; Figure S4: CAFs show a trend towards increase max migration speed, as compared to NF; Figure S5: No differences in the proliferation of HCC1143 cancer cells or fibroblasts in 2D co-cultures with HCC1143 with NF 3045N and CAF 2804T fibroblasts; Figure S6: No differences in the viability of HCC1143 cancer cells or fibroblasts in 2D co-cultures with HCC1143 with NF 3045N and CAF 2804T fibroblasts; Figure S7: Co-culture with fibroblasts in 3D does not change the viability of HCC1143 cancer cells, and the viability of CAF and control fibroblasts is similar in 3D; Figure S8: Cancer-associated fibroblasts induce the proliferation of cancer cells in 3D co-culture; Figure S9: Representative images of collagen I and hyaluronan distribution in 2D co-culture; Figure S10: HAS2 siRNA treatment decreases the hyaluronan content of CAF-derived ECM; Figure S11: HAS2 siRNA treated CAFs produce a less coherent ECM; Figure S12: Loss of hyaluronan in CAF-derived extracellular matrix does not change the proliferation of HCC1143 cells; Figure S13: Loss of hyaluronan in CAF-derived ECM result in less spread and more elongated HCC1143 cells; Text S1: FIJI Macro for spheroid image pre-processing and extraction of cell positions; Text S2: Python script to calculate the nearest neighbor distances between cell centroids within spheroids.
