# Supplementary material for: Cancer-Associated Fibroblasts Move and Interact More with Triple-Negative Breast Cancer Cells and Stimulate Their Proliferation in a Hyaluronan-Dependent Manner
Source: Cells. 2025 Oct 23;14(21):1663. doi: 10.3390/cells14211663 (PMC12606758; doi:10.3390/cells14211663)
Supplement: Supplementary file 1 [file cells-14-01663-s001.zip › cells-3875779-supplementary.pdf]

Supplementary Materials

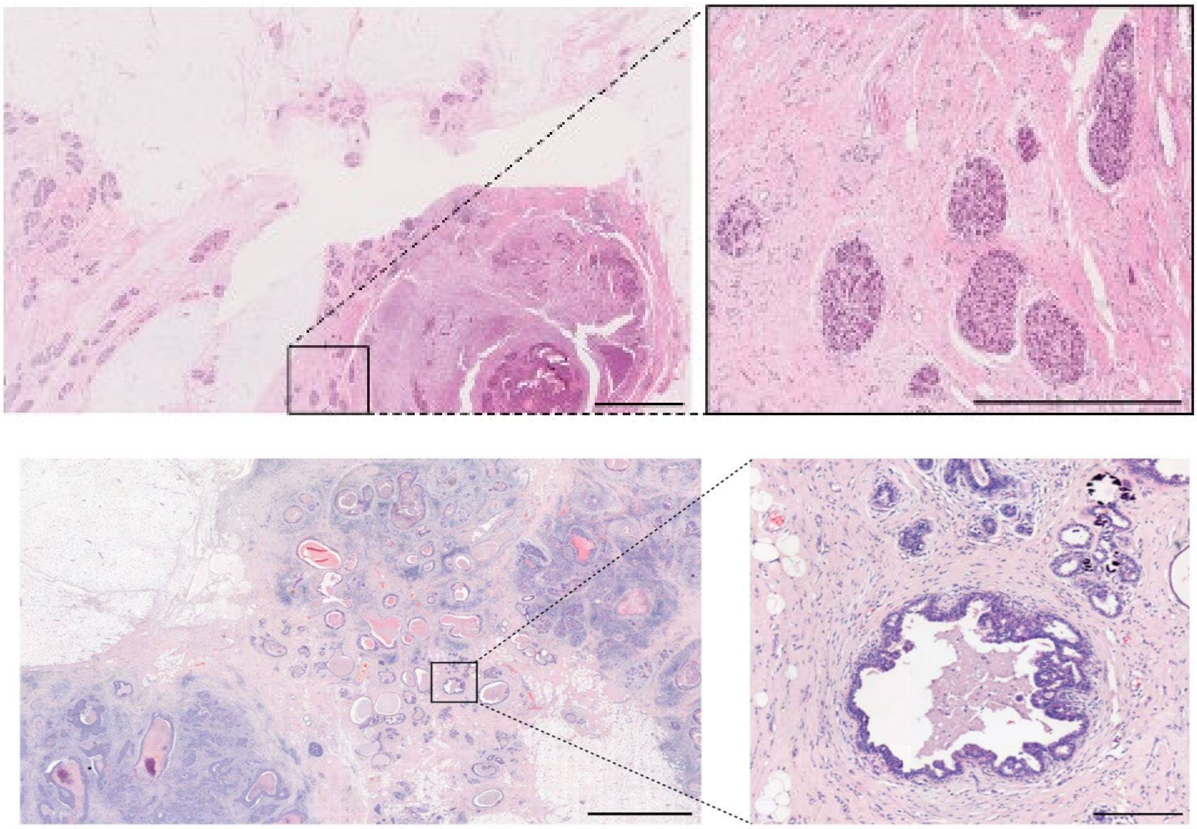

Supplementary Figure S1. Representative histological section of triple negative breast cancer. TNBC 2804T (top) or CAF 2262T (lower panel), sections stained with Haematoxylin and Eosin, with low resolution images (left) and magnified areas (right) showing circumscribed cancer cell clusters surrounded by abundant fibrous connective tissue with a compacted extracellular matrix and few scattered myofibroblasts and inflammatory cells (right). Scale bars, top left, 3 mm; top right, 1 mm; lower left, 2 mm, lower right, 0,2 mm. The staining was performed, and the images produced by Breast Cancer Now.

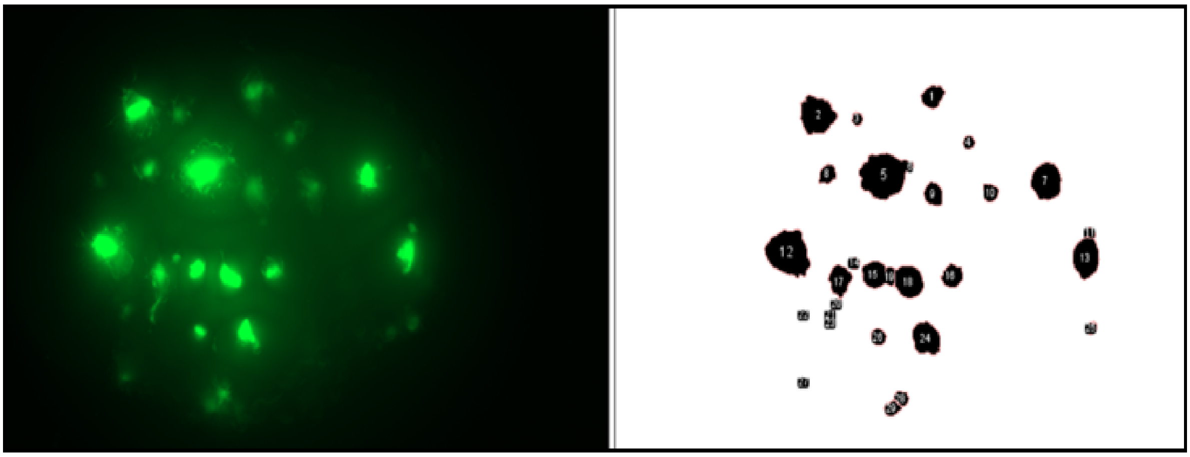

Supplementary Figure S2. Image acquisition and cell position extraction using FIJI. Representative example of signal from one channel (left), and the correspondent binary mask (right) showing the centroids (numbered) which allowed extraction of coordinates.

Supplementary Text S1. FIJI Macro for spheroid image pre-processing and extraction of cell positions. Two-dimensional positions of cells within the spheroid were extracted by preprocessing immunofluorescence-stained spheroid images. A binary mask was generated to identify individual cell centroids using the macro detailed, below.

```
run("Set Measurements...", "area mean center display redirect=None decimal=3");

setOption("ScaleConversions", true);

run("8-bit");

run("Z Project...", "projection=[Max Intensity]");

run("Duplicate...", "title=binary duplicate channels=1");

run("Duplicate...", " ");

run("Subtract Background...", "rolling=100");

setAutoThreshold("Huang dark");

setThreshold(30, 255, "raw");

setOption("BlackBackground", false);

run("Convert to Mask");

run("Minimum...", "radius=5");

run("Maximum...", "radius=5");

run("Watershed");

run("Analyze Particles...", "size=10-Infinity show=Centroids display");
```

Supplementary Text S2. Python script to calculate the nearest neighbor distances between cell centroids within spheroids. Pixel coordinates of cell centroids were converted to micrometer based on the pixel size (Image > Properties > Pixel width) in FIJI. A nearest neighbor distance analysis was calculated in Python by constructing a KDTree based on the scipy.spatial.KDTree algorithm. The input dataset is expected to include a column labeled Spheroid, and columns X and Y containing centroid coordinates.

```
import pandas as pd

from sklearn.neighbors import KDTree

introduce_data_path = input("path_file.ods ")

df = pd.read_excel(introduce_data_path, engine="odf")

distances_nn = []

for _, group in df.groupby('Spheroid'):
            coordinates = group[['X', 'Y']]

            tree = KDTree(coordinates)

            distances, _ = tree.query(coordinates, k=2)

            distances_nn.extend(distances[:, 1])
```

```
distances_nn = pd.Series(distances_nn)

print(distances_nn.head())
```

Note: The input file path (introduce_data_path) must be manually specified for each dataset analyzed.

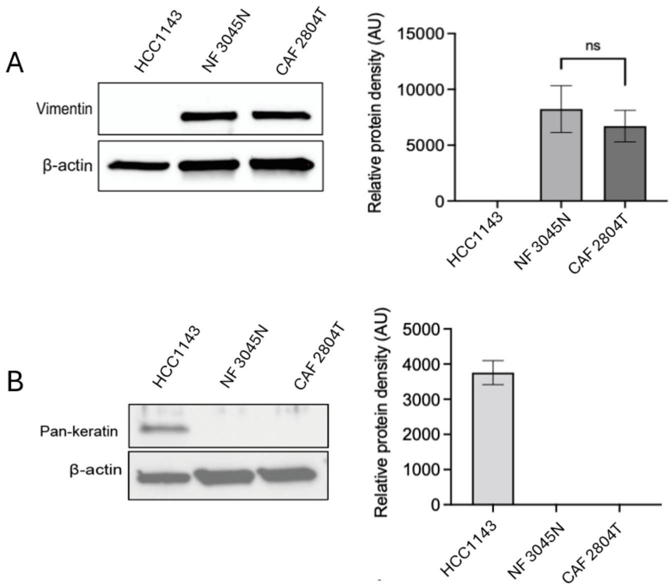

Supplementary Figure S3. Western blot characterisation of fibroblast and myofibroblast markers. (A) Representative western blot and quantification of HCC1143, NFs 3045N and CAFs 2804T and β-actin loading control. (B) Representative western blots and quantification of HCC1143, NFs 3045N and CAFs 2804T and β-actin loading control. Quantification shows the mean +/- SEM of ≥ 3 independent experiments. The data was analysed using an unpaired t-test. * = p < 0.05

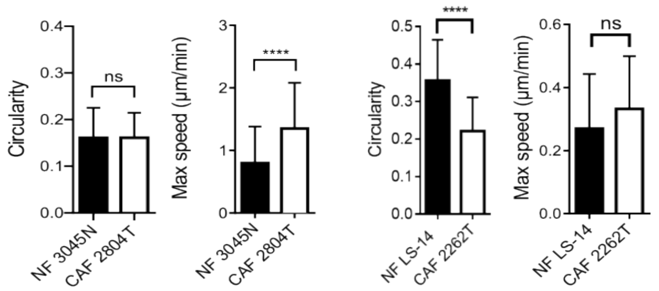

Supplementary Figure S4. CAFs show a trend towards increase max migration speed, as compared to NF. Quantification max speed and circularity. Graphs show mean ± SEM of 3 independent experiments for CAFs 2804T n ≥ 139 cells per condition The data was analysed using a Mann-Whitney test. * = p > 0.05, **** = p > 0.0001.

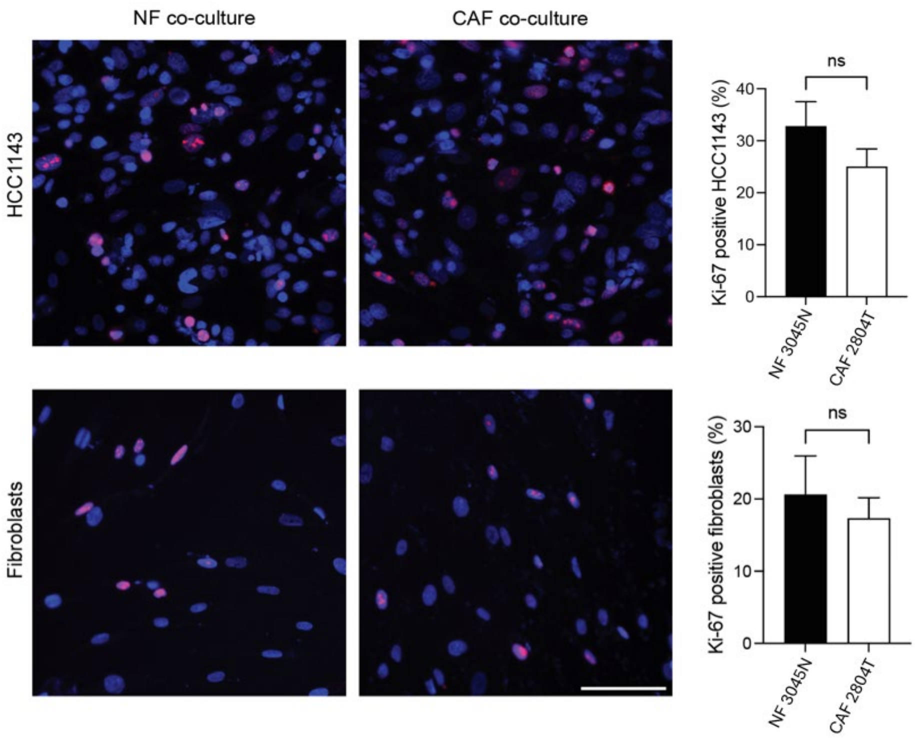

Supplementary Figure S5. No differences in the proliferation of HCC1143 cancer cells or fibroblasts in 2D co-cultures with HCC1143 with NF 3045N and CAF 2804T fibroblasts. Representative images of nuclei (blue) and Ki-67 (red) of HCC1143 and fibroblasts after 72 hours in 2D co-culture. Scale bar: 100 µm. Quantification shows the mean +/- SEM of at least three independent experiments with ≥ 6 islets and n ≥ 425 cells per condition, data analysed using unpaired t-tests.

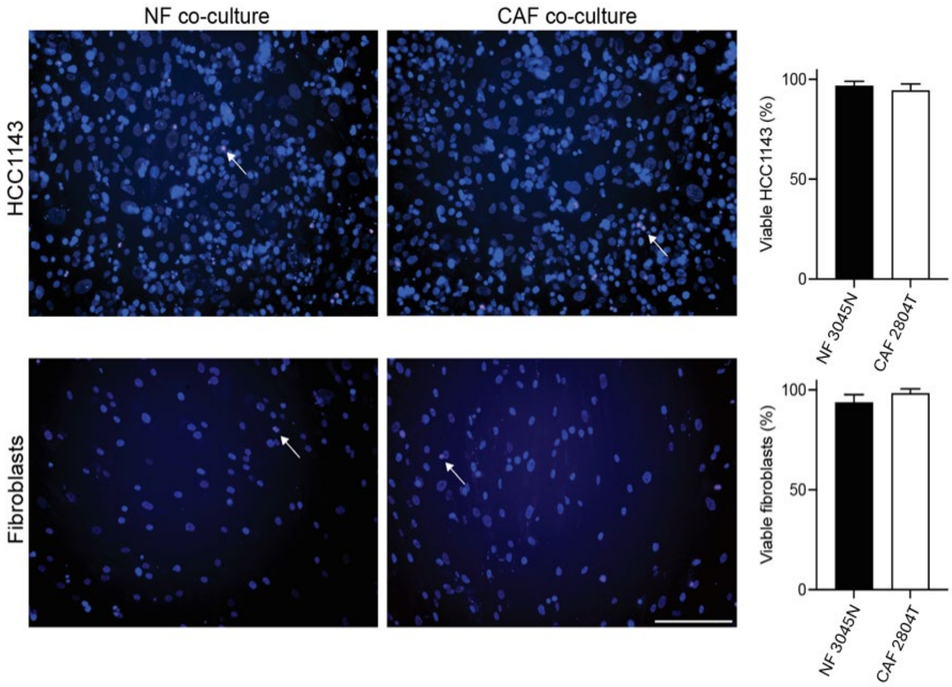

Supplementary Figure S6. No differences in the viability of HCC1143 cancer cells or fibroblasts in 2D co-cultures with HCC1143 with NF 3045N and CAF 2804T fibroblasts. Representative live images of HCC1143 and fibroblasts in 2D co-culture, showing Hoechst stain (blue) and propidium iodide stain (red). The white arrows indicate examples of dead cells in each panel. Scale bar: 200 µm. Quantification shows the mean +/- SEM of 3 independent experiments with ≥ 8 islets and n ≥ 849 cells total per condition. The data was analysed using a Mann-Whitney test.

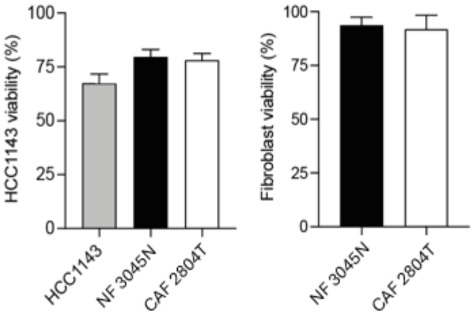

Supplementary Figure S7. Co-culture with fibroblasts in 3D does not change the viability of HCC1143 cancer cells, and the viability of CAF and control fibroblasts is similar in 3D. (A) the viability of HCC1143 in mono-culture or co-culture spheroids with CAF 2804T or NFs 3045N, and **(B)** fibroblast viability in co-culture. Quantification shows the mean +/- SEM from at least independent experiments with a total of n ≥ 23 spheroids per condition. HCC1143 viability was analysed with a one-way ANOVA with multiple comparisons, and fibroblast viability was analysed with an unpaired t-test.

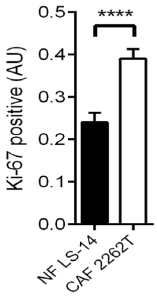

Supplementary Figure S8. Cancer-associated fibroblasts induce the proliferation of cancer cells in 3D co-culture. Ki67 positive HCC1143 cancer cells co-cultured in 3D with either NF LS-14 (black) or CAF 2262T (white) fibroblasts.

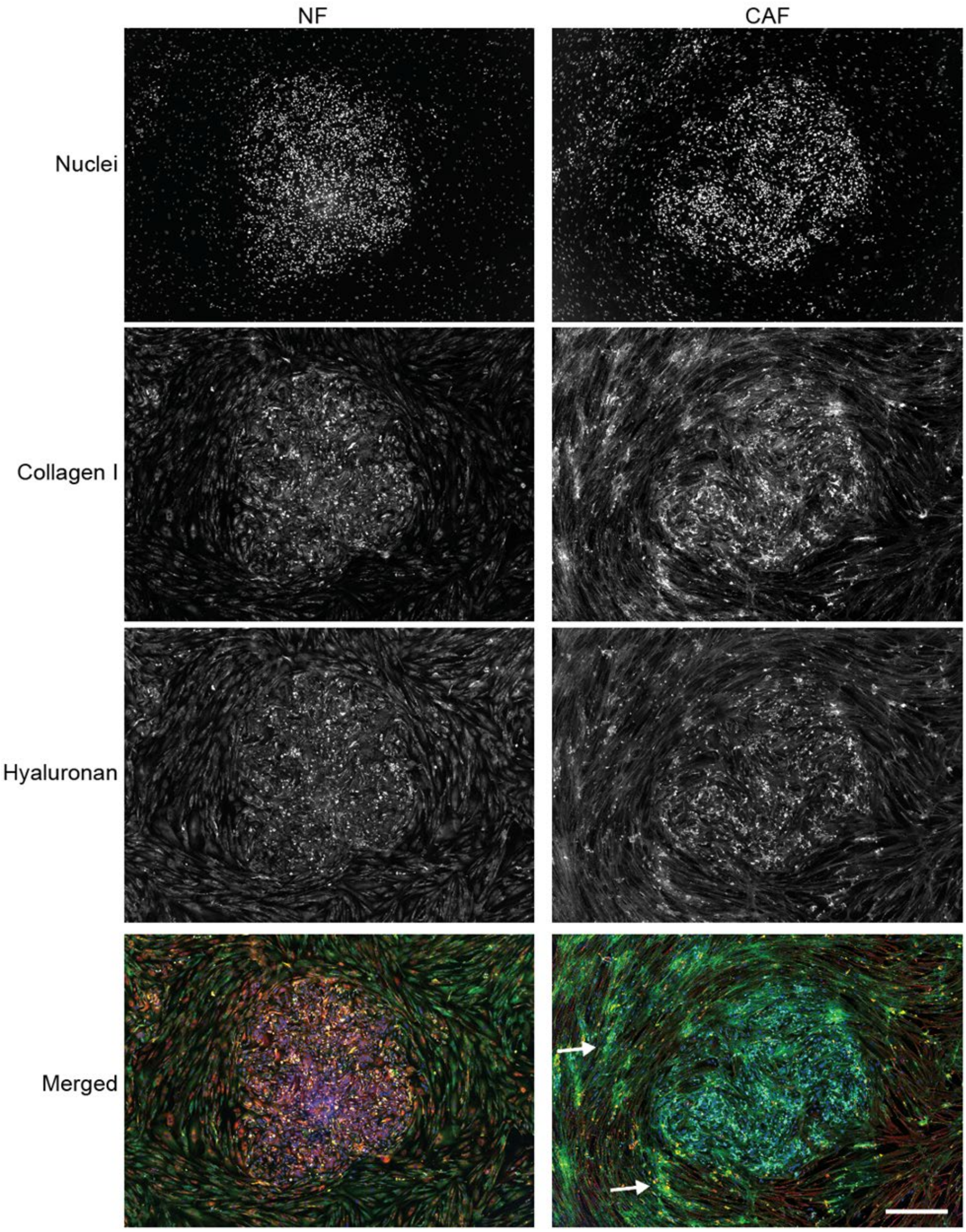

Supplementary Figure S9. Representative images of collagen I and hyaluronan distribution in 2D co-culture. The merged image shows nuclei (blue), collagen I (red) and hyaluronan (green) in NF 3045N and CAF 2804T. The white arrows indicate extracellular hyaluronan. Scale bar: 500 µm.

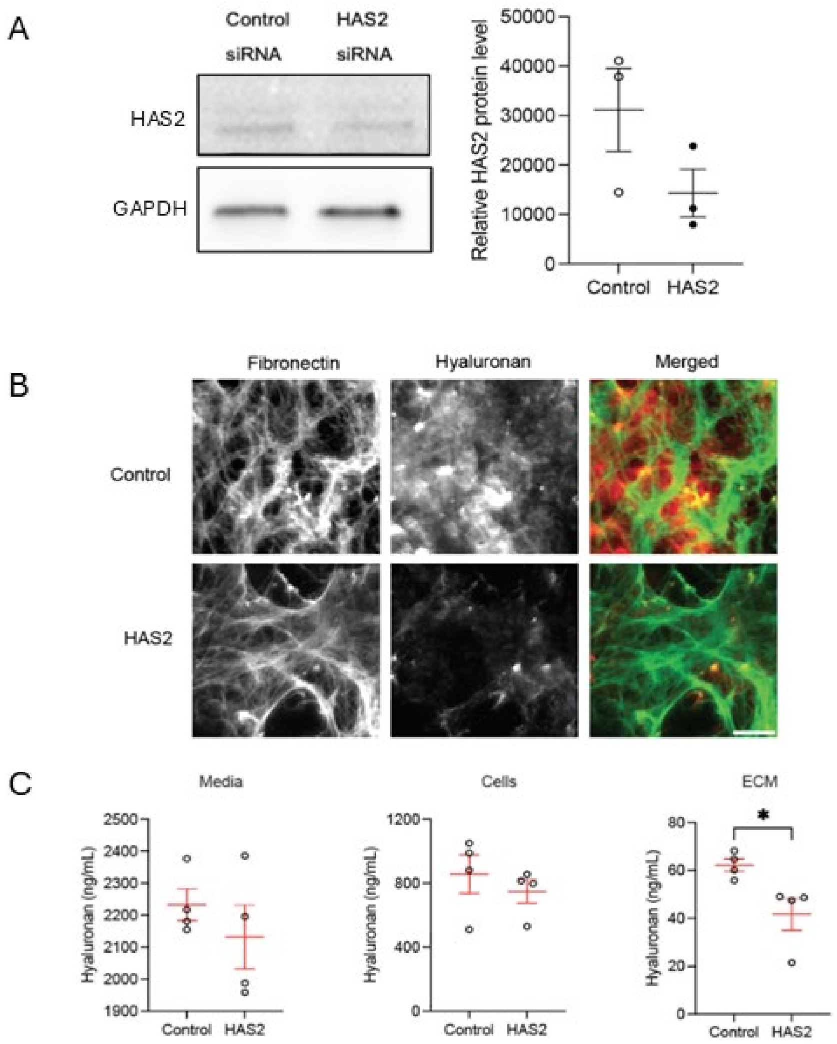

Supplementary figure S10. HAS2 siRNA treatment decreases the hyaluronan content of CAF-derived ECM. (A) Representative western blot and quantification of HAS2 protein levels normalised to β-actin as the loading control. Quantification shows the mean +/- SEM. The dots represent 3 independent experimental repeats. (B) Representative images showing fluorescence staining of decellularized matrix produced by control or HAS2 siRNA treated CAFs, showing fibronectin (green) and hyaluronan (red). Scale bar: 20 µm. (C) Quantification of hyaluronan content in media, cells and ECM from control or HAS2 siRNA treated CAFs. Quantification of hyaluronan shows the mean +/- SEM of 4 independent experiments. The dots represent individual replicates. Data was analysed using a Mann-Whitney test. * = $p < 0.05$.

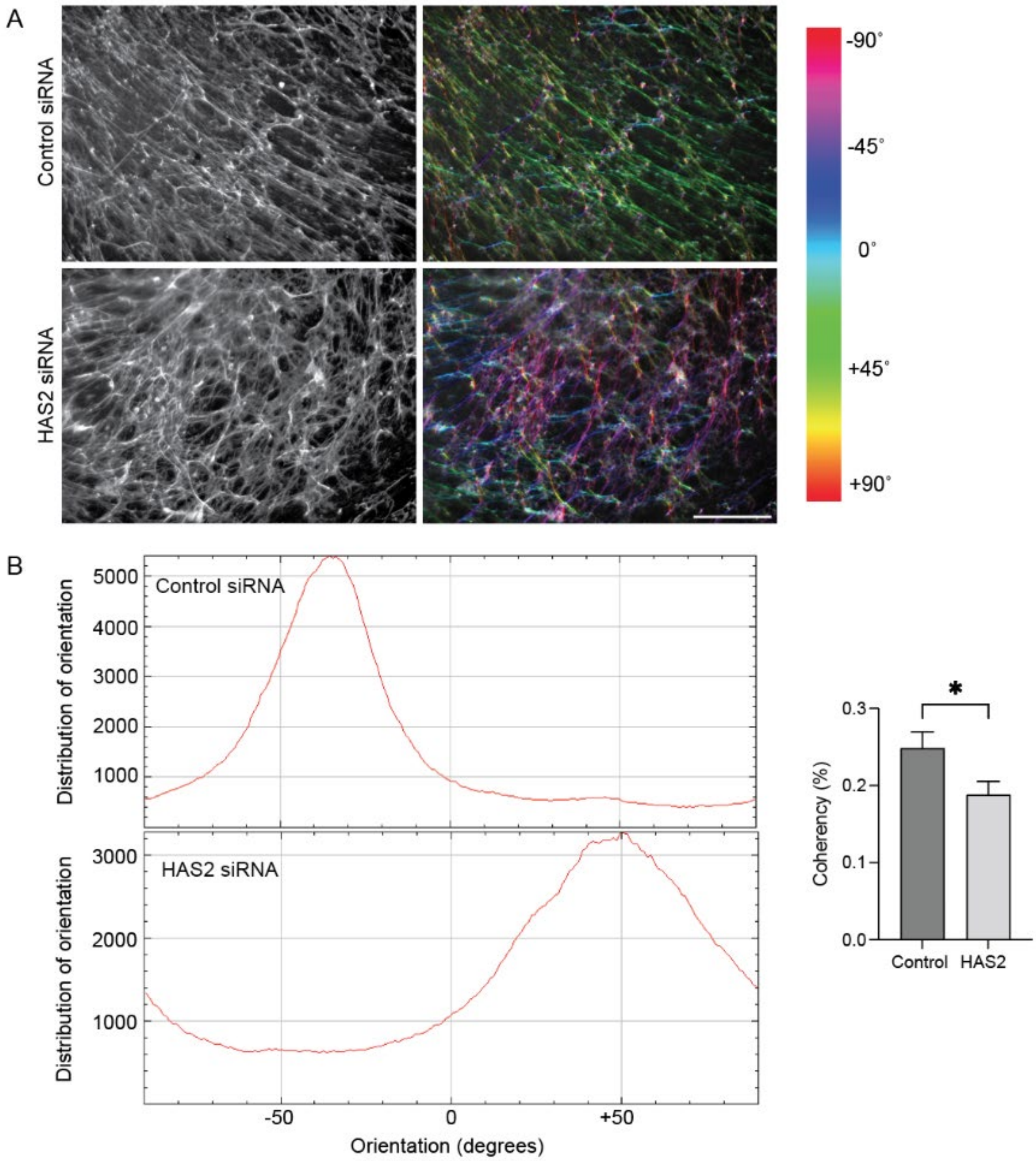

Supplementary figure S11. HAS2 siRNA treated CAFs produce a less coherent ECM. (A) Representative images of ECM derived from CAF treated with control or HAS2 siRNA. Right column shows immunofluorescence staining of fibronectin in white, and images in the left column are colourised according to matrix orientation, as specified in the colour bar. **(B)** Representative graphs showing the distribution of fibre orientation from the images in (A). Quantification of distribution of orientation shows the mean +/- SEM of 3 experimental repeats. Data was analysed using an unpaired t-test. * = $p < 0.05$.

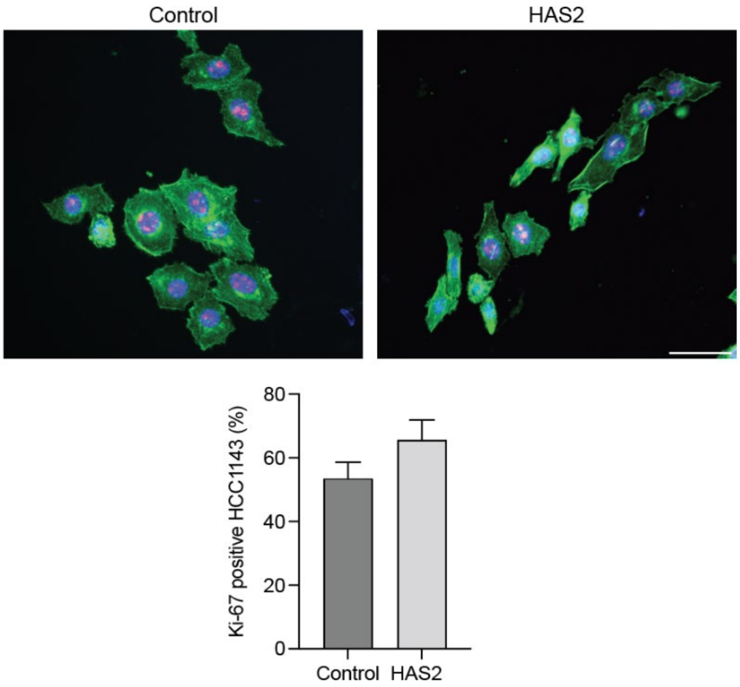

Supplementary Figure S12. Loss of hyaluronan in CAF-derived extracellular matrix does not change the proliferation of HCC1143 cells. Representative immunofluorescence images of HCC1143 cultured on control or HAS2 siRNA treated CAF-derived ECM, showing nuclei (blue), F-actin (green) and Ki-67 (red). Scale bar: 50 µm. Quantification of Ki-67 positive cells shows the mean +/- SEM. Data was obtained from 3 independent experiments with n ≥ 9 fields of view analysed per condition. Data was analysed using an unpaired t-test.

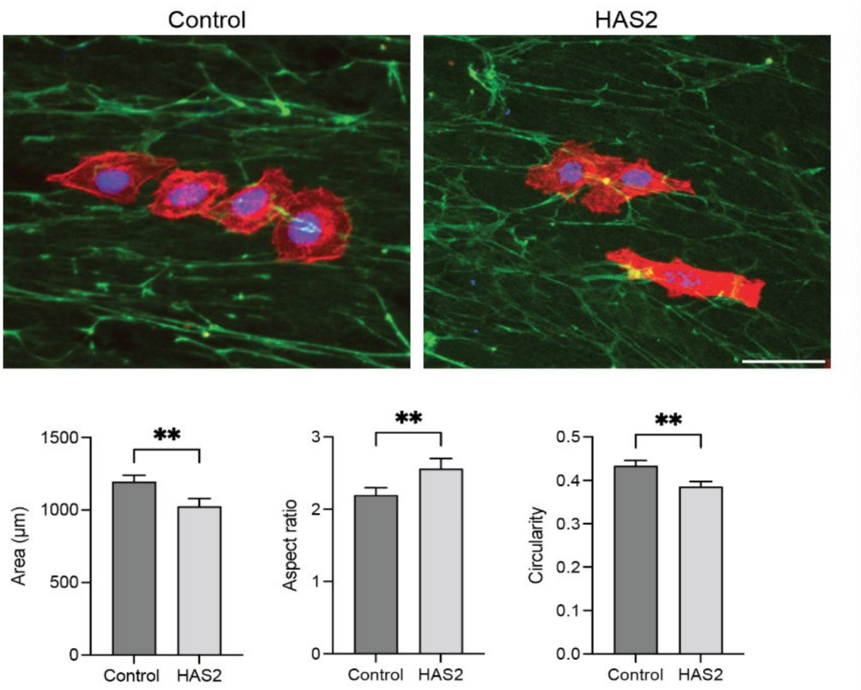

Supplementary Figure S13. Loss of hyaluronan in CAF-derived ECM result in less spread and more elongated HCC1143 cells. Top panel, representative images of HCC1143 cultured on HAS2 siRNA or control- treated CAF-derived ECM, showing nuclei (blue), fibronectin (green) and F-actin (red). Scale bar: 50 µm, with quantifications (lower panel). Data shows as the mean +/- SEM. Data was obtained from 3 independent experiments with n ≥ 125 cells per condition, shown as the mean +/- SEM, and analysed using a Mann-Whitney test. ** = p < 0.01.
